# Editorial: Latest trends in bio-inspired medical robotics: structural design, manufacturing, sensing, actuation and control

**DOI:** 10.3389/frobt.2025.1544097

**Published:** 2025-01-28

**Authors:** Yilun Sun, Houde Dai, Shuang Song, Angela Faragasso, Sara-Adela Abad Guaman

**Affiliations:** ^1^ Institute of Micro Technology and Medical Device Technology, Technical University of Munich, Garching, Germany; ^2^ Quanzhou Institute of Equipment Manufacturing, Fujian Institute of Research on the Structure of Matter, Chinese Academy of Sciences, Jinjiang, China; ^3^ School of Mechanical Engineering and Automation, Harbin Institute of Technology, Shenzhen, China; ^4^ FingerVision Inc., Tokyo, Japan; ^5^ Department of Mechanical Engineering, University College London, London, United Kingdom

**Keywords:** medical robotics, bio-inspired robotics, soft robotics, structural design, sensor, actuator, robot control

## 1 Introduction

Over the past few decades, robotic technologies have been widely introduced into different medical applications, such as surgical operation and rehabilitation engineering, to improve the efficiency and quality of medical treatment. However, those robots usually need to interact with humans and manipulate their complex structure and internal organs via small openings, which presents a big challenge for the current sensing, actuation and control strategies (Muscolo and Fiorini, 2023; Sun and Lueth, 2023b). To solve these problems, many researchers have introduced biologically inspired techniques into medical robots. For example, snake-like soft robots are used to achieve flexible bending motions in minimally invasive surgery (Burgner-Kahrs et al., 2015; Lin et al., 2024; Cianchetti et al., 2018; Ashuri et al., 2020; Sun et al., 2020; Sun and Lueth, 2023a), while insect-inspired exoskeleton robots can provide walking assistance to patients with disabilities (Shi et al., 2019; Yang et al., 2023; Liao et al., 2023).

In this Research Topic, we aim to present the latest developments and achievements of bio-inspired technologies for supporting the future research directions within the field of medical robotics, including structural design, modeling, manufacturing, sensing, actuation and control. As a result of the call for participation, seven papers were finally accepted and collected in this Research Topic.

## 2 Overview of the contents of the Research Topic

The first two articles are focusing on the structural design of robotic systems for medical robots. In the paper “*A compact motorized end-effector for ankle rehabilitation training*” by Wu et al. the authors presented the design and development of an end-effector ankle rehabilitation robot called CEARR to support range of motion ankle rehabilitation. The CEARR employed a bilaterally symmetrical structure with three degrees of freedom per side, driven by independent actuators, and integrated a real-time voluntary-triggered control (VTC) strategy using surface electromyography (sEMG) and torque signals to enhance rehabilitation outcomes. The proposed VTC strategy could be more cost-effective than neural-network-based algorithms, as it can be executed on a single microcontroller with fewer computational resources. In the paper “*Optimization and fabrication of programmable domains for soft magnetic robots: A review*” by Bacchetti et al. the authors reviewed the current state of the art of programmable magnetic soft robots, focusing on bio-inspired structural optimization and fabrication. The paper indicated that significant further developments of programmable magnetic soft robots could be achieved by increasing the computational power of novel optimization methods, combined with advances in computational resolution, material options and automation of fabrication methods.

The contribution in “*Novel bio-inspired soft actuators for upper-limb exoskeletons: design, fabrication and feasibility study*” by Zhang et al. analyzes the actuator design for medical robots. In that paper, two kinds of soft actuators were developed for upper-limb exoskeletons: the Lobster-Inspired Silicone Pneumatic Robot (LISPER) for the elbow and the Scallop-Shaped Pneumatic Robot (SCASPER) for the shoulder. Experimental results showed that, by using position control and gravity compensation mode, an upper-limb exoskeleton equipped with the proposed actuators can stably track the desired trajectory and maintain the desired position.

Other two contributions address the Research Topic of tactile sensor design for medical robots. The paper “*Validations of various in-hand object manipulation strategies employing a novel tactile sensor developed for an under-actuated robot hand*” by Singh et al. presented an opto-electronic-based tactile sensor, which was integrated into an under-actuated prosthetic hand (Prisma Hand II) to realize complex in-hand object manipulation. Based on the voltage value from the tactile sensor, deep learning methods were developed to calculate the grasping forces and torques for object manipulation. The paper “*Abraded optical fibre-based dynamic range force sensor for tissue palpation*” by Dawood et al. on the other hand, introduced a variable-stiffness dynamic range force sensor based on abraded optical fibre, which can be used to provide remote haptic feedback. By adjusting the stiffness of the sensor, the measurement range of touching force can be modified.

The last two articles are focusing on the motion control of medical robots. In the paper “*Integrating computer vision to prosthetic hand control with sEMG: Preliminary results in grasp classification*” by Wang et al. the authors investigated the feasibility of integrating sEMG signals with visual information to improve the accuracy of prosthetic hand control. Results showed that, during the early reaching phase, a higher accuracy of grasp pattern classification could be achieved with the integrated vision data. Based on this knowledge, more vision-based methods could be developed in the future to enhance the motion control accuracy of myoelectric prosthetic hands. In the paper “*Adaptive approach for tracking movements of biological targets: application to robot-based intervention for prostate cancer*” by Smahi et al. the authors presented a robotic system for Brachytherapy in prostate cancer treatment. By utilizing a deep learning framework based on Long Short-Term Memory (LSTM) networks and Convolutional Neural Networks (CNNs) to predict the position of prostate, the proposed system can precisely deliver the radioactive drug to the cancer tissues and hence, improve the patient experience in prostate cancer Brachytherapy.

## 3 Conclusion

The articles collected in this Research Topic provide a good demonstration of how bio-inspired techniques could improve the performance of medical robots. Despite the significant progress, several challenges still remain in the future development of bio-inspired medical robots. For instance, in soft medical robots, innovative solutions are needed to protect delicate electronic components from damage during large deformations of the robot body. Additionally, onboard computation for AI-based control of medical robots still faces limitations due to weight and power constraints. From this perspective, more collaboration between clinicians, roboticists, biologists and mechanical engineers should be encouraged in the future to further promote the development of medical robotics.
